# An assessment of the impact of extraction and digestion protocols on multiplexed targeted protein quantification by mass spectrometry for egg and milk allergens

**DOI:** 10.1007/s00216-019-01816-z

**Published:** 2019-05-28

**Authors:** Chiara Nitride, Jørgen Nørgaard, Jone Omar, Hendrik Emons, María-José Martínez Esteso, Gavin O’Connor

**Affiliations:** 1 European Commission, Directorate-General Joint Research Centre, Retieseweg 111, 2440 Geel, Belgium; 2 Present Address: School of Biological Sciences, Division of Infection, Immunity and Respiratory Medicine, Manchester Academic Health Science Centre, Manchester Institute of Biotechnology, The University of Manchester, 131 Princess Street, Manchester, UK; 3 Present Address: Fachbereich 3.2 Biochemie, Physikalisch-Technische Bundesanstalt, Bundesalle 100, 38116 Braunschweig, Germany

**Keywords:** Food allergens, Mass spectrometry, Protein, Extraction, Quantification

## Abstract

The unintentional presence of even trace amounts of certain foods constitutes a major hazard for those who suffer from food allergies. For many food industries, product and raw ingredient surveillance forms part of their risk assessment procedures. This may require the detection of multiple allergens in a wide variety of matrices. Mass spectrometry offers a possible solution for the quantification of multiple allergens in a single analysis. The capability of MS to quantify many peptides from a complex protein digestion is well known. However, a lack of matrix certified reference materials has made the optimisation of extraction and digestion conditions for multiplexed allergen quantification difficult to assess. Here, we report a systematic study, using preliminary screening followed by a Design of Experiments approach, to find the optimal buffer and digestion conditions for detecting milk and egg protein markers in a model processed food matrix. Five of the most commonly used buffers, two chaotropic reagents and two reducing reagents were assessed for the optimal extraction of multiple protein markers. While the choice of background buffer had little impact, the use of chaotropic and reducing reagents showed significant benefits for the extraction of most proteins. A full factorial design experiment was applied to the parameters shown to have a significant impact on protein recovery. These studies suggest that a single optimal set of extraction conditions enabling the quantitative recovery of all proteins is not easily achieved. Therefore, although MS is capable of the simultaneous quantification of many peptides in a single run, greater consideration of protein extraction is required before these are applied for multiplex allergen quantification in food matrices.

**Electronic supplementary material:**

The online version of this article (10.1007/s00216-019-01816-z) contains supplementary material, which is available to authorized users.

**Figure:**
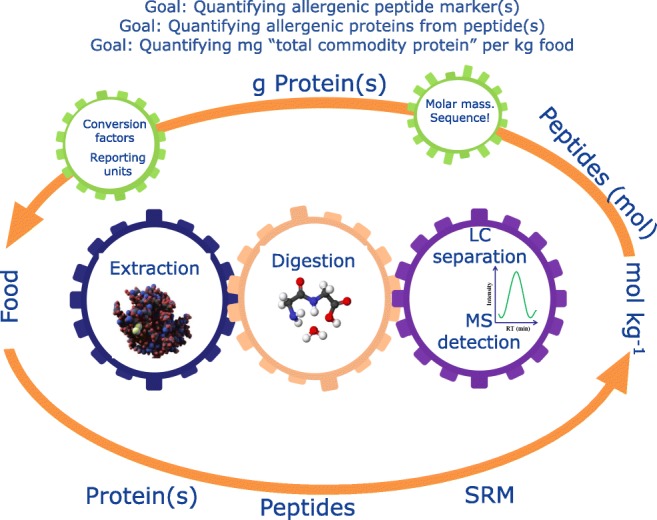
Graphical abstract

## Introduction

Food allergy is caused by the adverse reaction of the immune system in a sensitive individual toward a specific food. Currently, there is no clinical treatment or cure for those suffering from a food allergy. Affected people must be careful about what they eat and rely on appropriate food labelling to avoid exposure to the offending food. EU legislation, namely Regulation (EU) No 1169/2011 [1], requires food manufacturers to list all information regarding food ingredients on the product label and to highlight the presence of a specific group of food ingredients that may trigger food allergies and intolerances. The unintentional presence of an allergen represents a food safety hazard which is particularly difficult to manage. Food allergens may enter the food supply chain at any time during storage, handling and packaging as a consequence of cross-contact within processing lines. Food business operators should adopt risk management procedures to prevent, eliminate, or reduce the risk of contamination to acceptable levels, thus avoiding the occurrence of food allergy incidents and food product recalls.

A quantitative risk assessment measures the impact of a specific hazard [2] as a combination of reference doses and exposure. Such an approach is currently used by the VITAL® (Voluntary Incidental Trace Allergen Labelling) risk assessment procedures [3]. The thresholds for allergenic proteins in food are based on clinical data (Oral Food Challenge) and are indicators of the action levels, expressed as the total protein of the allergenic food (mg), below which only the most sensitive allergic subjects may react. Precautionary allergen labelling (PAL), such as “may contain” statements, should only be used after performing a quantitative risk assessment, the results of which suggested a demonstrable and significant risk of allergen contamination at levels above the action level [4]. Analytical methods help to support the validation and verification of the risk assessment outcomes, providing quantitative measurements of the allergenic ingredient in processed food [5]. Any method measuring allergen markers must express their results in quantities and measurement units that are comparable with other methods. For use in the risk assessment procedures, this requires the allergen content to be expressed in milligrammes of allergen protein per kilogramme of food [6]. The feasibility of mass spectrometry-based methods for the detection and quantification of allergens has been proven [7–10]. A considerable number of studies have been dedicated to the optimisation of mass spectrometry-based methods for the detection of egg and milk proteins using peptides from a single “marker” protein from each food [7–15]. One of the major advantages of mass spectrometry is its ability to sequentially detect marker peptides enabling the multiplexed analysis of many allergens. Several food matrixes have been addressed: biscuits [7–10, 16]; breakfast cereals; infant food [11]; and wine [12–15, 17], using a diverse range of mass spectrometry platforms and methods. In particular, a targeted mass spectrometry approach, known as selective reaction monitoring (SRM), was successfully applied for the detection of multi-allergen targets [7–13]. This method has the advantage of selectively detecting marker peptides, from specific allergen proteins, in complex background matrices and allows the measurement of protein abundances that differ by four to five orders of magnitude. Peptides are quantified based on monitoring specified precursor peptide-to-product ion transitions. For absolute quantification, the method of isotope dilution mass spectrometry (IDMS) is often applied [18]. This requires the measurement of the relative response of the natural peptide, generated by proteolytic cleavage of the target protein, and its heavy isotopically labelled form, added at a known quantity to both the standards and the samples [19]. This method of measuring the peak area ratios reduces systematic errors in peptide quantification [9–12]. However, as the internal standard (isotopically labelled peptide) is added as the free peptide, it does not redress any deficiencies in protein extraction or release of the peptide from its precursor protein during digestion [20]. Therefore, the largest contributor to the measurement uncertainty and/or bias when labelled internal peptide standards are used is expected to derive from the extraction and digestion steps in the analysis. It is essential to ensure that total extraction and complete digestion of the proteins occur as only this will enable a true assessment of the concentration of the allergen present [16].

To circumvent this issue, expression of a bovine ^15^N-*α*_*S1*_-casein protein, to correct for extraction and recovery after baking, was suggested by Newsome et al. [21]. Although encouraging recovery factors were reported, the labelled protein was spiked into the biscuits after the baking process; therefore, the unprocessed labelled protein may not possess the possible heat-induced modifications resulting in different extraction and digestion behaviours being experienced by the natural and labelled protein.

Despite the fact that most of the published reports [6–14] select the same marker proteins and proteotypic peptides, from milk and egg, the extraction and digestion protocols differ significantly, even when the same food matrix is analysed. Unlike in ELISA [22], no systematic investigation concerning the optimisation of extraction and digestion protocols for food allergen quantification has been performed when using MS.

The aim of the study presented in this paper was the optimisation of the extraction and digestion protocols for the quantitative removal of milk and egg proteins from a model processed food matrix. The quantitative targeted proteomic pipeline was separated into three critical components that contribute to the trueness and uncertainty of the final answer: extraction, digestion and SRM analysis. The first step consists in the selection of the marker peptide and the development of the SRM method, including proteotypic peptides from five milk allergenic proteins and two egg allergenic proteins. We investigated the impact of different extraction conditions on the SRM peptide signal response, which is the chemical entity measured in a bottom-up proteomic experiment. The peptide abundance reflects the variation of the performance of the extraction of the proteins from the food. The food matrix studied was a biscuit incurred with the allergenic proteins prior to heat treatment, thereby mimicking commercially available products. An experimental design or Design of Experiments (DoE) approach was used for the optimisation of the extraction and digestion conditions. The main advantage of DoE is that the maximum information of the evaluated system is obtained by the minimum number of experiments. Moreover, it allows the study of many variables at the same time providing information on the interaction among the variables, which cannot be achieved using a one variable at a time approach. By this simultaneous optimisation of the multiple peptide responses, an optimal compromise has to be reached between them to have one method that suits all. ANOVA on the final optimised conditions allowed the assessment of the impact of three analytical stages (extraction, digestion and measurement) on the overall variability. To the best of our knowledge, this is the first quantitative study showing the extent to which peptide response can vary depending on the extraction and digestion conditions chosen.

## Materials and methods

### Materials

Standard proteins of αS-casein (C6780), β-casein (C6905), κ-casein (C0406), β-lactoglobulin A (L7880), ovalbumin (A5503), lysozyme (L6876), [Glu1]-fibrinopeptide B human (Glu-Fib) (F3261-1MG), myoglobin from equine heart (M1882), peptide standards (angiotensin II human (A9525-1MG), Met-Arg-Phe-Ala acetate salt (M1170-1MG), bradykinin acetate salt (B3259-1MG), angiotensin I human acetate salt hydrate (A9650-1MG) were purchased from SIGMA (St. Louis, MO, USA).

The reagents used for protein extraction and digestion including acetone, hydrochloric acid, sodium hydroxide, urea, guanidine-HCl, sodium phosphate buffer (PBS), ammonium bicarbonate (AmBic), trizma base, boric acid, sodium tetraborate, triethylammonium hydrogen carbonate buffer (TEAB), dithiothreitol (DTT), iodoacetamide (IAA) were purchased from SIGMA (St. Louis, MO, USA). Trifluoroacetic acid (TFA) and mercaptoethanol (BME) were purchased from Merck (Darmstadt, Germany).

Solvents including ULC-grade acetonitrile (ACN), ULC-grade water and 99% formic acid (FA) were purchased from Biosolve (Valkenswaard, The Netherlands).

Trypsin Gold-Mass Spec Grade was purchased from Promega (Madison, USA).

HyperSep™ C18 (200 mg, 3 mL) Cartridges were obtained from Thermo Scientific (Biopolymers Ulm, Germany).

The following heavy-isotope-labelled peptides, whereby all the carbon and nitrogen atoms in the n-terminus lysine or arginine were replaced with 99% ^13^C and ^15^N, YLGYLEQLLR* (αS1), FALPQYLK* (αS2), LYAEER* (Ova), FESNFNTQATNR* (Lys), were purchased from JPT (JPT Peptide Technologies GmbH, Germany).

Two batches of biscuits, one blank and one containing 5000 mg of protein ingredient per kilogramme of biscuit, were prepared following a common recipe for cookies by the Institute of Food Research in Leatherhead, UK, in the frame of the iFAAM project. The baking temperature was 150 °C for 9 min. Milk and egg ingredients were purchased from BIOSERVICE Zach GmbH (Austria). The Spray Dried skimmed milk powder (09G010) contained 34.8% *w*/*w* milk protein and the egg white powder (1-022161VOIB) contained 71.6% *w*/*w* egg protein. The biscuit recipe also included hazelnut, peanut and walnut flours; however, these proteins were not targeted by the analysis outlined here. A detailed description of the recipe is given in the Electronic Supplementary Material (ESM; S1).

### Operating conditions for the selective reaction monitoring by mass spectrometry

Five allergenic proteins from milk (αS1 casein—Bos d 9, αS2 casein—Bos d 10, β-casein—Bos d 11, κ-casein—Bos d 12, β-lactoglobulin—Bod d 4) and two from egg white (ovalbumin—Gal d 2, lysozyme—Gal d 4) were selected as markers that would indicate the presence of the food ingredients. Protein sequences were digested in silica using the predicted cleavage sites for trypsin, and the proteotypic marker peptides were selected based on specific characteristics. The applied criteria for peptide marker selection included the following: the uniqueness of the peptide for the marker protein, determined using the MS-Homology blast tool of protein prospector (http://prospector.ucsf.edu/prospector/mshome.htm), searching the Swissprot and the NCBInr databases; a lack of peptides containing cysteine and natural post-translational modifications (PTMs); the resulting tryptic peptides containing between 5 and 12 amino acid residues [23].

A Xevo TQ-S triple quadrupole (QqQ) mass spectrometer (Waters, Manchester, UK) equipped with a Trizaic ion source was used to acquire the SRM data for this study. Standard proteins were digested with trypsin (50 mmol L^−1^ Ambic, pH 8; 1:50, E:S) and the released peptides were directly infused into the QqQ using a syringe pump at 1.5 μL min^−1^. The optimised source parameters used were as follows: capillary voltage 3.5 kV; cone voltage 25 V; source temperature 100 °C; cone gas flow 50 L h^−1^; collision gas flow 0.11 mL min^−1^. Nitrogen (99.998% purity, L’Air liquid Belgie, Liège, Belgium) and argon (99.999% purity, L’Air liquid Belgie, Liège, Belgium) were used as the cone and collision gases, respectively. The QqQ was first operated in a scanning mode to define the optimum conditions for the precursor ion signals. Only charge states between + 2 and + 4 were considered when optimising the source conditions as these were responsible for the most intense peptide signals from the digested milk peptides. Subsequently, the mass spectrometer was operated in product ion scanning mode, where the top y- and b-ions were ranked in increasing order of intensity and the top 6 were selected for further collision energy (CE) ramping optimisation. Seven different collision cell voltages, between 8 and 22 V in increments of 2 V, were studied.

The mass spectrometer was coupled to a nano Acquity-UPLC system (Waters, Manchester, UK). The chromatographic separation of the peptides was performed on a C18 reversed-phase ionKey system (BEH C18, 130 Å, 1.7 μm, 150 μm × 50 mm). The solvents used were 0.1% FA in water (solvent A) and 0.1% FA in ACN (solvent B). The sample injection volume was 2 μL using a full loop injection. A dual pump reverse flush trapping configuration was applied. The sample was loaded on a trapping column (Symmetry 300 C18, 5 μm, 300 μm × 50 mm) and an isocratic flow of 1% B over 2 min with a flow rate of 10 μl min^−1^ was applied. The trapping column was then back flushed in line with the ionKey at a flow of 2 μL min^−1^ utilising the following gradient: 1–5% B over 0.5 min, 5–33% B over 15.5 min, 33–99% B over 2 min, 99% B for 3.5 min, 99–1% B in 1 min and finally 1% B for 4.5 min to allow the column to re-equilibrate.

Two to three transitions for each peptide, preferably where the selected m/z of the product was greater than that of the precursor, were included in the final SRM protocol. The dwell time was fixed at 50 milliseconds for each measured transition. The SRM transitions were separated into different timed events resulting in a minimum of 12 points per chromatographic peak for each transition monitored.

The data acquisition and the data elaboration were performed using MassLynx and TargetLynx software from Waters. The chromatographic peaks were integrated automatically, and a peak area list was generated for each peptide from each sample. For each natural and isotopically labelled peptide pair, the peak area ratio natural/labelled was calculated. For peptides where no isotopically labelled analog peptide was available, a universal isotopic standard peptide was used. The universal internal standard peptide chosen was FALPQYLK* from αS2-casein as it eluted in the middle of the gradient.

A system suitability test (SST), including an MS1 scan and a product ion scan, was performed before every set of analysis to verify that the method performs within its specifications (retention time, *m/z*).

### Protein quantification

The RCDC™ (reducing agent and detergent compatible) protein assay (BioRad) was used to determine the total protein concentration in the sample extracts. Blank samples, carried through all stages of the manipulation, were used as blanks or zero references.

### Screening: extraction and digestion

Five buffering systems—ammonium bicarbonate (AMBIC) [9, 10], tris-buffer (TRIS) [6–8], phosphate buffer saline (PBS) [24], triethylammonium bicarbonate buffer(TEAB) [12], borate buffer saline (BBS) [25]—two chaotropic agents [26, 27]—urea and guanidine—and two reducing agents—dithiothreitol(DTT) and mercaptoethanol (BME)—were screened. Separate buffer solutions of AMBIC, TRIS, PBS, TEAB and BBS were prepared using MilliQ water (18 MΩ cm^−1^), at 50 mmol L^−1^ concentration and pH 8.0. The chaotropic agents, urea and guanidine, were added to separate aliquots of the buffer solutions, resulting in a final concentration of 5 mol L^−1^of each. The reducing agents were added to separate aliquots of the urea AMBIC and urea BBS buffer solutions so that DTT was 50 mmol L^−1^ and the BME was 2% (*v*/*v*). A graphical representation of the screening approach buffer solutions is provided in Fig. S1 (see ESM). A total of 35 different extraction combinations were investigated in triplicate.

One gram of the ground biscuit was solubilised in the extraction buffer (1:15, *w*:*w*), mixed vigorously for 1 min and sonicated in a cold bath for 5 min. The extraction was carried out for 6 h at 22 °C. The solution was centrifuged at 9500 rpm for 45 min at 22 °C and the supernatant was collected. The extracted proteins were precipitated with four volumes of 80% cold acetone overnight at − 27 °C. The solutions were centrifuged at 9500 rpm for 30 min at 4 °C and the acetone was discarded. The precipitate was washed twice with 3 mL of cold acetone and precipitated at − 27 °C for 1 h each time. The acetone was evaporated using a nitrogen purge at room temperature; the precipitates were resuspended in 2.5 mL of 10 mmol L^−1^ DTT, 3 mol L^−1^ urea, 50 mmol L^1^ AmBic at pH 8.2. Two hundred fifty microlitres of the protein extract were diluted with 25 mmol L^−1^ AmBic to yield a final concentration of 1 mol L^−1^ urea, which is below the accepted threshold for trypsin digestion. The horse myoglobin (SIGMA M1882) was used as a quality control of the trypsin digestion across the extraction buffers studied. One hundred microlitres of stock solution (14.2 nmol) were added to each extract and the proteins were reduced (10 mmol L^−1^ DTT, 1 h at 37 °C) and alkylated (50 mmol L^−1^ IAA, 0.5 h at 22 °C in the dark). Trypsin was added at an enzyme to substrate ratio of 1:50 and the digestion was carried out for 16 h at 37 °C, while applying a constant gentle shaking. The digestion was stopped by adding formic acid to a *v*/*v* of 0.1%.

### Experimental designs: extraction and digestion

The developed method used the Design of Experiments to organise and suggest the experiments in conjunction with a response surface methodology for the further analysis of the results obtained. The Unscrambler® X software (v10.3, CAMO, Trondheim, Norway) was used for DoE and further data treatment.

In both optimisations, extraction and digestion, the same procedure was followed, whereby a preliminary full factorial design (FFD) was performed to identify which of the studied parameters or variables had a significant effect on the extraction and digestion processes. The FFD was followed by a central composite design (CCD), in which the statistically significant parameters from the FFD were optimised. The optimised variables for the extraction and digestion can be observed in Tables 1 and 2, respectively.

**Table 1:** Experimental factors and evaluated levels for the FFD and CCD of the extraction

Experimental factors	FFD			CCD				
	**−**	0	+	**−**	**−**	0	+	++
Temperature (°C)	7	22	37	0	2	7	12	14
pH	6.5	7.5	8.5					
Time (h)	3	6	9					
AmBic (mM)	25	50	75					
DTT (mM)	25	50	75					
Urea (M)	3	6	9	2.8	3.7	5.8	7.9	8.7

**Table 2:** Experimental factors and evaluated levels for the FFD and CCD of the digestion

Experimental factors	FFD			CCD				
	**−**	0	+	**−**	**−**	0	+	++
Time (h)	2	5	8	9	11.25	13.5	15.75	18
AmBic (mM)	25	50	75	0	6.25	12.5	18.75	25
DTT (mM)	25	50	75	0	6.25	12.5	18.75	25
ACN (%*)	0	5	10					
DMSO (%*)	0	5	10	0	6.25	12.5	18.75	25

*Final volume

### Preparation of peptides for SRM analyses

Trifluroacetic acid was added to the digests to a final *v*/*v* of 0.1%. Isotopically labelled peptides were added to the solution at mass fractions corresponding to 50 mg kg^−1^ of the total protein content of the target allergenic food ingredient. All the digests were diluted ten times to yield a final concentration of organic solvent below 1% to enable optimal isolation on the SPE cartridge. Peptides were isolated on HyperSep™ C18 Cartridges (200 mg) (Thermo Scientific, Biopolymers, Ulm, Germany). The columns were activated with methanol (5 column volumes (CV)) and equilibrated with 0.1% TFA, 5% ACN in water (5 CV). After loading the sample, the column was washed with 0.1% FA, 5% ACN in water (10 CV); eluted in 0.1% FA, 60% ACN in water (10 CV). Peptides were concentrated using a nitrogen evaporator at ambient temperature and then diluted with 0.1% FA in MilliQ water up to a final volume of 1 mL. Since the isotopically labelled peptides have identical chemical and physical behaviour to the allergen marker peptides, any possible loss of a peptide marker due to the analytical procedures post digestion will be accounted for, as the ratios of natural to labelled peptide peak areas were monitored. All solutions were spiked with a standard peptide, Glu-1-Fibrinopeptide B (Glu-Fib), at a concentration of 50 ng mL^−1^ before the mass spectrometry analysis. This was used purely to assess the stability of the MS platform over the analysis timescale.

### Rate of digestion

Proteins were extracted and digested using the optimised protocols. The peptide release rate was evaluated, by monitoring the natural to labelled peptide ratios using the SRM method, over a range of 36 h, during which 14 sample points were collected at 0, 0.5, 1, 2, 3, 4, 6, 8, 10, 12, 22, 26, 30 and 34 h. Two enzyme-to-substrate ratios were considered: 1:50 and 1:100, where the trypsin was added only at time zero, the beginning of the incubation. The multiple addition of the enzyme was also studied. 1:50 was added at time zero and after 22 h of digestion—to a final 1:25 (E:S) ratio; 1:100 was added at time zero, after 6, 12 and 22 h of digestion—to a final 1:25 (E:S) ratio.

### Nested design

The repeatability of the final method was assessed using a two-factor fully nested experiment design. Three separate extracts of the 5000 mg of the total ingredient protein/kg ground biscuits were performed on the same day. Each extract was digested in triplicate. Four additions of the enzyme (1:100, E:S) were performed every 3 h over the digestion time of 12 h. Three replicate measurements were performed per digested sample.

## Results and discussion

The specific purpose of this study was to investigate the influence of the extraction and digestion conditions on the SRM signal response for peptides selected as suitable markers for the quantification of “total” egg and milk protein. The final aim was the development of a multiplexed quantitative method of analysis. The extraction and digestion protocols were optimised for baked biscuits and the repeatability of the extraction and digestion were studied via a two-way nested ANOVA study design.

The SRM method development consisted of four main steps: (1) selection of allergenic marker proteins from egg and milk; (2) selection of proteotypic peptides by in silico digestion of the proteins; (3) method development by hydrolysing the standard proteins with trypsin; (4) method refinement by analysing hydrolysed crude proteins extracted from the biscuits.

The protein markers for cow’s milk (*Bos domesticus*) were as follows: α-S1-casein, α-S2-casein, β-casein, κ-casein and β-lactoglobulin. The protein markers for hen’s eggwhite (*Gallus gallus*) were as follows: ovalbumin and lysozyme. Twenty-seven peptides were selected as candidate proteotypic peptides according to conventional criteria [21].

The specificity and uniqueness of the peptides were established by comparing the peptide sequences against the online protein databases. Only four and two peptides were found to be unique for cow’s milk and hen’s egg, respectively (ESM Table S1). However, while most of the candidate peptides were unique for milk and egg, they were not species-specific. Two of the target peptide sequences from milk proteins and one from egg proteins were found in other non-milk- and egg-derived proteins but were not excluded at this stage of the screening.

The optimisation of the chromatography and the mass spectrometry operating conditions was performed using trypsinised standard proteins. The final SRM comprised the *m/z* of the precursor (Q1) and the *m/z* of two or three product ion transitions (Q3) (ESM Fig. S2). The optimal instrumental conditions are listed in Table S2 (see ESM).

The crude extract (6 mol L^−1^ urea, 50 mmol L^−1^ Ambic, pH 8) of the biscuit proteins was hydrolysed (1:50, E:S) and the released peptides were analysed by SRM. The selected peptides were refined based on the signal intensity and specificity of each transition (interference). The peptides AMKPWIQPK and ITVDDK-αS2; TPEVDDEALEK and IDALNENK-βLg; EAMAPK and EMPFPK-βCN were not included in the final method because the signal intensity was extremely low and the chromatographic resolution was poor.

The influence of different buffer composition and conditions was studied in order to maximise protein extraction. Preliminary screening of the extraction conditions was performed using five buffers, two chaotropic agents (guanidine hydrochloride and urea) and two reductants (DTT and BME). The extraction buffers were selected according to those commonly reported in the literature.

The extraction performance was evaluated at the protein level by RCDC. Changing the buffers did not reveal any major impact on protein extractability (ESM Fig. S3, panel a). By adding the chaotropic agent alone, the extractability increased by a factor of 7, while in combination with the reductant, it further increased by a factor of 3 (ESM Fig. S3, panel a and c). Urea was the best performing chaotropic agent and increased the yield of extraction significantly by denaturing proteins. The RCDC assay only provides information relating to the total protein concentration. It does not deliver detailed information about specific proteins. The information at the peptide level was acquired by SRM analysis of the trypsinised extract. An influence of the extraction buffer on trypsin digestion efficiency cannot be excluded, even after the precipitation and clean-up of the proteins. The AmBic, the TEAB and the BBS were the extraction buffers that showed the greatest compatibility with trypsin digestion. The combined use of a chaotropic and reducing agent increased the peak area of the peptides from β-lactoglobulin by a factor of 10, for lysozyme by a factor of 13 and for ovalbumin by a factor of 4 (ESM Figs. S4 to S7). The observed increase depended on the side chain interactions (i.e. disulfide bridges) and the location of the peptide in the protein sequence. Similar increases in signal, when using the reducing reagents, were not observed for the alpha and beta casein proteins (ESM Fig. S6). The reductants break the gluten network helping the release of the proteins and also break the disulfide bonds making the protein more accessible to the enzyme. Therefore, it is not surprising that the reductants are not significantly improving extraction and digestion for the alpha and beta caseins since they do not contain cysteine disulfide bridges. In contrast, the κ casein does contain a disulfide bond and experience an increase in extraction on the inclusion of the reducing reagents. The performance between the two reducing agents studied was not significantly different for most peptides using a *t* test (*P* = 95%) (ESM Figs. S6-S7). Therefore, DDT was selected for the further studies as this was more compatible with current working practices.

The screening evidence suggested that there was little observable difference between the background buffer studied when looking at the total protein content (ESM Fig. S3); the use of chaotropic agents was beneficial, with urea, resulting in a greater extraction efficiency than guanidine for most proteins; the use of a reducing reagent was beneficial especially for proteins with disulphide bridges, with little observable difference between DDT and BMR. Based on this screening evidence, AmBic, urea and DTT were chosen as the best performing components of the extraction buffer. Moreover, these components are ESI-MS-compatible. Consequently, the sample digest solution can be directly injected into the LCMS for SRM analysis, minimising the sample handling which can lead to peptide loss.

As mentioned previously, a two-stage DoE was used to optimise the extraction and digestion protocols with the aim of creating a single multi-allergen method. A FFD was employed to study the major influencing variables of the extraction and digestion steps. Subsequently, a CCD was used to optimise the statistically significant variables of each of the steps.

Following the optimisation of the experimental conditions for screening (amount of sample, choice of extraction buffer, etc.), the optimisation of the extraction and the digestion steps were achieved in sequential experiments. In the first step, the extraction was optimised by fixing the digestion parameters, i.e. a generic digestion method was used while varying the extraction conditions. In the second step, the digestion was optimised using the fixed optimal extraction conditions observed previously. In the FFD of the extraction, six variables (pH, temperature, time, AmBic concentration, urea concentration and DTT concentration) were studied at two levels. The variables studied and their respective levels are summarised in Table 1. In addition, a central point was included in the design at which all factors were kept at the mean value of the two levels. This was used to estimate the analytical precision of the experiments. Thirty-two experiments were performed in a randomised order. The total protein extraction efficiency was determined prior to trypsin digestion using the RCDC™, while the protein-specific information was obtained by monitoring the peak area ratios of the individual peptides after digestion. Generally, the total protein yield of extraction is the only response that is optimised in protein extraction experiments, but in this particular case, where the goal is to quantify peptides as a surrogate for the intact protein, the study of the individual peptide peak areas becomes essential.

**Fig. 1:**
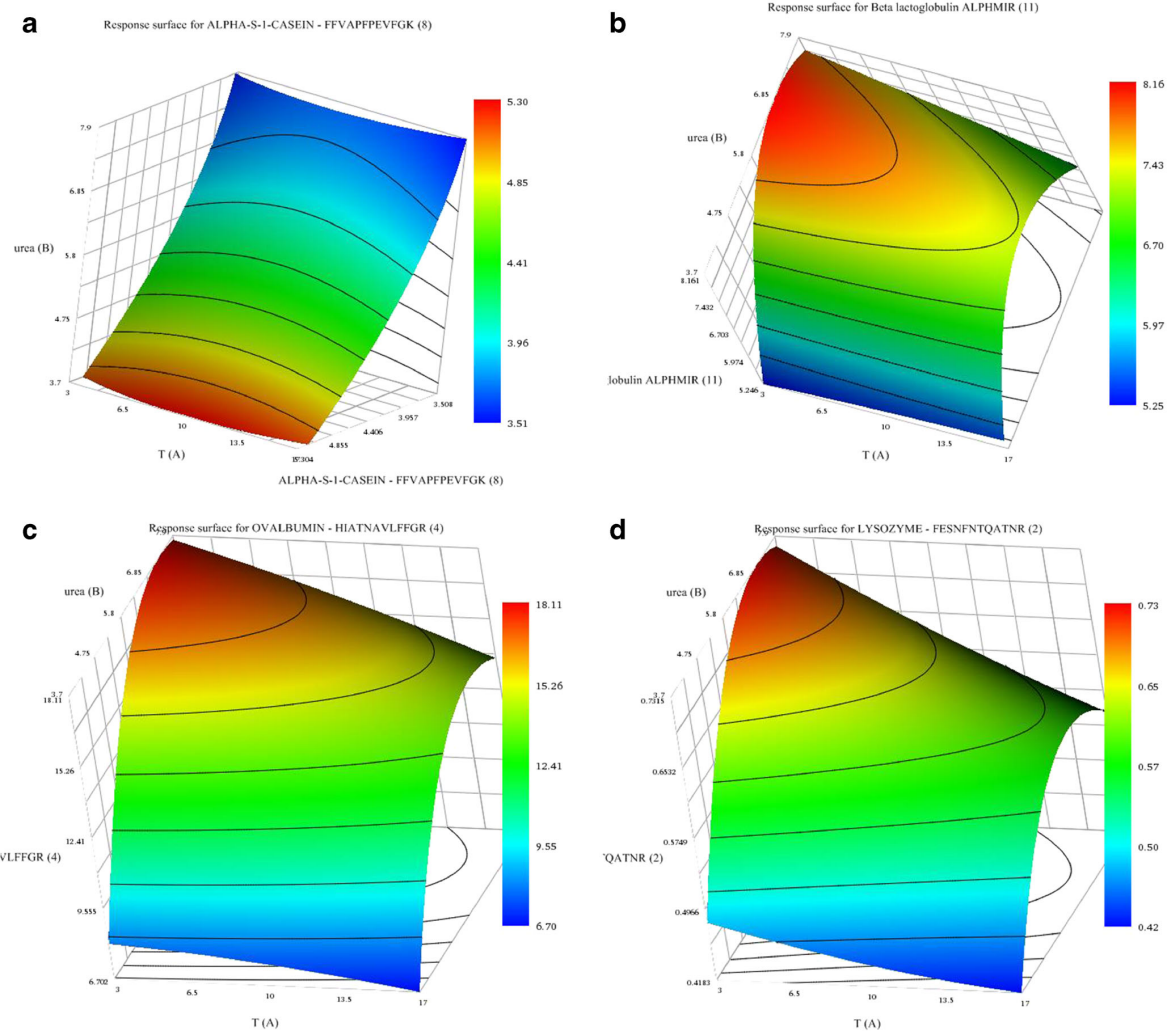
Surface plots showing the effects of the urea concentration and the extraction temperature on the peak area of four prototypic peptides (*p* level < 0.05). **a** α S1 CN-FFVAPFPEVFGK. **b** β LG-ALPMHIR. **c** Ova-HIATNAVLFFGR. **d** Lys-FESNFNTQATNR

According to both the multilinear regression (MLR) and the partial least square (PLS) models, constructed using Unscrambler® software, only the temperature and the urea concentration were statistically significant (*p* level < 0.05) (ESM Table S3). In the case of the PLS, variables with a regression coefficient higher than 0.2 are typically considered significant, and in the MLR approach, the analysis of variance (ANOVA) of the regression resulted in the same conclusion. The other variables were fixed at the central values with the exception of the extraction time, which was fixed at the minimum to save time. The statistically non-significant variables were fixed at 50 mmol L^−1^ AmBic, 50 mmol L^−1^ DTT, pH 8 and 3 h of extraction. A central composite design (CCD) was performed to optimise the statistically significant variables from the FFD (urea concentration and temperature). Fourteen experiments were performed, including six central points at 5 levels (Table 1). The evaluation of the response surfaces showed that urea concentration and temperature of extraction influenced the majority of the peptide responses (ESM Table S4). Figure S10 (see ESM) shows the response surface plot of the urea concentration versus the temperature for total protein concentration. The highest total protein values were observed when the two variables were at their lowest values (see Table 1). The SRM analysis of the peptides, released after trypsin digestion, revealed contrasting behaviours (Fig. 1 and ESM Fig. S10). The maximum peak area ratio for the signals of the released peptides from caseins was achieved when both urea concentration and temperature were low. On the other hand, the peak area of ovalbumin and lysozyme target peptides was maximised when the concentration of urea was higher and the temperature was lower. Based on the response surfaces of the peptides, the final conditions for the extraction step were fixed at 50 mmol L^−1^ AmBic, 5 mol L^−1^ urea, 50 mmol L^−1^ DTT, pH 8, temperature 10 °C.

The high concentration of urea and the higher temperature denatures the protein structure, thus solubilising and preventing precipitation and aggregation. However, these harsh conditions can chemically modify lysine residues and protein N-terminus by non-enzymatic carbamylation making the peptide undetectable by the SRM analysis [28, 29]. The need to dilute the urea concentration to a level compatible with trypsin activity may also lead to protein refolding and precipitation [30].

A universal extraction and digestion protocol from a biscuit matrix is the main goal of this work, even if it may not be applicable for all food allergens, food matrices and processing conditions. To optimise protocols for the multiplex analysis of egg and milk allergens, a compromise has to be reached as the different proteins exhibit different optimal extraction behaviour. Residues of the extraction buffer can influence the trypsin activity, even after several cleaning steps. Minimal sample handling helps to reduce protein losses, as already stated. Therefore, the cold acetone precipitation was eliminated at this stage and the proteins were digested directly.

For the digestion, we wish to evaluate the response from each individual peptide peak area ratio (natural:labelled). According to both MLR and PLS models, all the parameters considered for the FFD were statistically significant (ESM Table S5). The digestion performance was better with lower concentrations of AmBic and DTT, the absence of ACN and a higher concentration of DMSO. Moreover, digestion was not complete after 8 h; thus, the time was extended in the CCD. From the CCD it can be concluded that the concentrations of AmBic (mmol L^−1^) and DMSO (%) were the factors influencing the majority of the peptide responses (ESM Table S6).

**Fig. 2:**
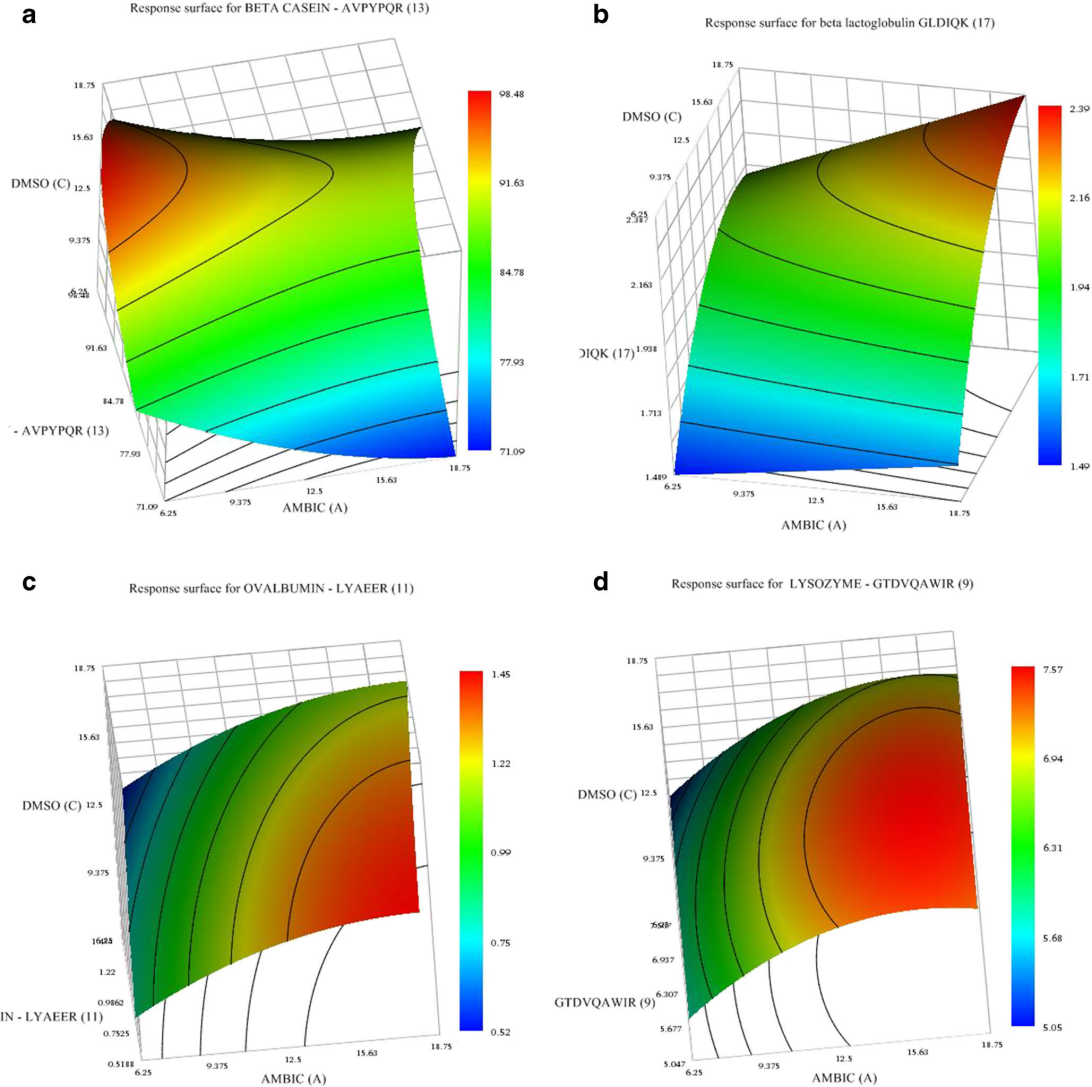
Surface plots showing the effects of DMSO and AmBic concentration on the peak area of four prototypic peptides after digestion (*p* level < 0.05). **a** β CN-AVPYPQR. **b** β LG-GLDIQK. **c** Ova-LYAEER. **d** Lys-GTDVQAWIR

The time of digestion was significant for peptides deriving from αS2CN (ALNEINQFYQK), ovalbumin (LYAEER) and lysozyme (GTDVQAWIR). This can be related to the protein structure as well as the location of the peptide within the protein. Higher AmBic concentrations resulted in the maximum peak area for all SRMs. The DMSO concentration had a quadratic effect on the signal generated by the two β-casein-derived peptides and the ovalbumin-derived peptide LYAEER, indicating that the optimal concentration of DMSO is in the middle of the experimental range. All the other peptides were not influenced (*p* level > 0.05). The concentration of DTT (mmol L^−1^) did not significantly affect the peak area; therefore, it was set at 5 mmol L^−1^ for the optimised method. The response surfaces demonstrating the effects of the significant factors on the peak area ratio are shown in Fig. 2 and ESM Fig. S11. For all the peptides studied, a maximum was observed after 18 h of digestion; thus, the time needed to carry out the extraction was fixed at 18 h. In summary, the optimised conditions used for the digestion were fixed at 25 mmol L^−1^ AmBic, 5 mmol L^−1^ DTT, 10% DMSO with a digestion time of 18 h.

**Fig. 3:**
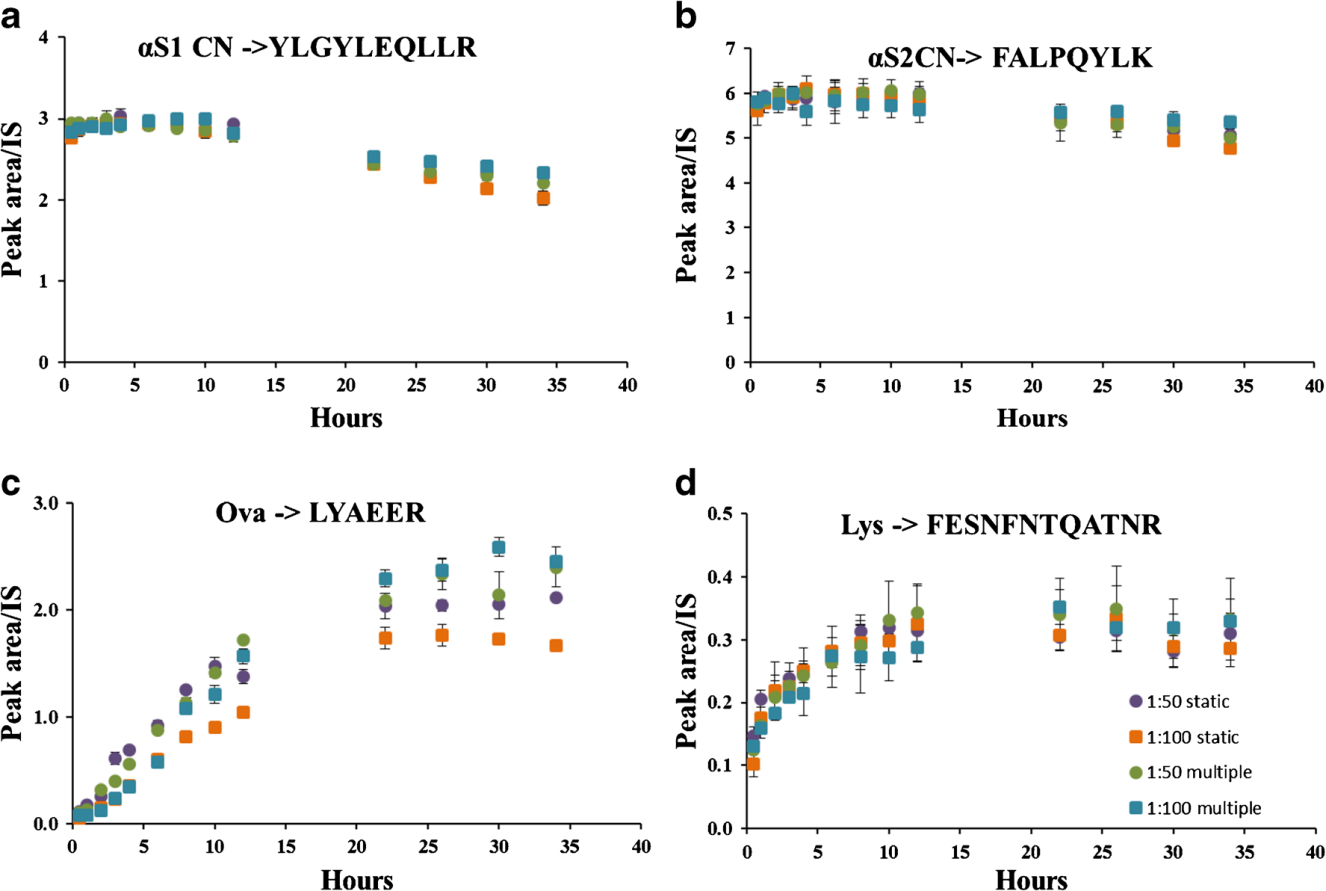
Profile of marker peptides released under optimised digestion conditions over 35 h of digestion. The addition of trypsin was static and multiple. Static addition of trypsin was performed only at time 0 h at two E:S ratios—1:50 and 1:100. Multiple additions of trypsin were performed at equidistant intervals of time to a final 1:25 (E:S) ratio—1:50 (E:S) ratio was added at time 0 and time 22 h; 1:100 (E:S) ratio was added at time 0, 6, 12 and 22 h. Error bars represent the standard deviation of 3 replicate measurements. **a** α S1 CN-YLGYLEQLLR. **b** α S2 CN-FALPQYLK. **c** Ova-LYAEER. **d** Lys-FESNFNTQATNR

A peptide acting as a reliable quantifier must be fully released from its constituent protein. The peptide has to be in a stable form in the final digestion solution such that its molar amount is representative of the moles of protein present in the extract. Any degradation or unreleased peptide will result in the incorrect determination of the concentration of protein initially present in the sample. To investigate the rate of protein digestion, a time course experiment was performed. Several aliquots of the extract were digested, and at each time point of the digestion, an aliquot was taken for analysis. The effects of enzyme concentration and digestion time for four peptides from the different egg and milk proteins are shown in Fig. 3 and ESM Fig. S12. It can be clearly observed that for peptides derived from the egg proteins, lysozyme and ovalbumin, a digestion time in excess of 16 h was required to reach a plateau for the peptide concentration (Fig. 3 and ESM Fig. S12). This plateau effect can be indicative of the total digestion of the protein present. Hence, the amount-of-substance concentration of the peptide would represent the concentration of the initial protein. The effect of digestion time on the measured milk peptides is markedly different from those of egg (Fig. 3 and ESM Fig. S12). The αS1 casein peptides are completely released in a relatively short digestion time and can be observed to decay after 16 h. This may indicate the degradation and/or the chemical modification of the peptide once it is released from the parent protein. The two β-casein peptides and one β-lactoglobulin peptide were adversely affected by both enzyme concentration and time (ESM Fig. S12, panel f, i, j). For most proteins, a digestion time of 12–20 h was optimal, but again, a careful selection of the peptides that are stable and fully released from their constituent protein is essential for accurate quantification.

The overall effect and the relative uncertainty contribution from the extraction and digestion steps were investigated using a simple two-factor full nested design. For this, three separate protein extracts were each digested three times and each digest was analysed in triplicate. The digests were analysed in a random order on the LCMS and statistical outliers were removed before performing the ANOVA. An advantage of performing such an experiment is that the individual contributions of the extraction, digestion and LCMS analysis to the overall measurement variability can be quantified. This is informative when designing the method validation plan as the factors influencing the overall uncertainty could be better controlled and/or repeated to assess overall precision. The overall precision of the measurement and the individual contributions from the repeatability of extraction, digestion and measurements for the milk and egg white peptides are reported in Table 3. As expected, the measurement precision for the β-casein and the β-lactoglobulin peptides, which showed rapid degradation over the digestion time, was poor. Where present, the addition of isotope-labelled peptide internal standards improved the precision of peptide measurements greatly.

**Table 3:** Overall relative variance for the repeat analysis of milk- and egg-derived peptides. Contributions expressed as % relative from extraction, digestion and LCMS peak area ratio measurements

Protein	Peptide	Source of variability	SS	MS	Total CV%	Variance % of total
αS1	YLGYLEQLLR*	Extraction	0.146	0.073	5	14.0
		Digestion	0.280	0.047		67.8
		Ratio measurement	0.069	0.004		18.2
	FFVAPFPEVFGK	Extraction	2.29666E+12	1.14833E+12	10.3	0.8
		Digestion	6.6312E+12	1.1052E+12		41.5
		Ratio measurement	6.30122E+12	3.50068E+11		57.7
αS2	VIPYVR	Extraction	65,911,263,315	32,955,631,658	6.4	2.0
		Digestion	1.22099E+11	20,349,773,838		0.0
		Ratio measurement	9.14193E+11	1.01577E+11		98.0
	FALPQYLK*	Extraction	0.546	0.273	5.6	0.0
		Digestion	1.694	0.282		75.4
		Ratio measurement	0.356	0.040		24.6
	NAVPITPTLNR	Extraction	6.12179E+11	3.0609E+11	45	0.4
		Digestion	9.24282E+11	1.54047E+11		0.0
		Ratio measurement	6.32202E+13	7.02446E+12		99.6
	ALNEINQFYQK	Extraction	21,969,405,628	10,984,702,814	10.9	0.0
		Digestion	88,508,367,129	14,751,394,522		42.0
		Ratio measurement	83,726,462,791	4,651,470,155		58.0
βCN	VLPVPQK	Extraction	3.62137E+13	1.81068E+13	40	17.7
		Digestion	6.57953E+13	1.09659E+13		81.4
		Ratio measurement	6.92243E+11	38,457,965,145		0.9
	AVPYPQR	Extraction	6.2232E+12	3.1116E+12	37.2	0.0
		Digestion	2.13986E+13	3.56643E+12		92.8
		Ratio measurement	1.61913E+12	89,951,794,843		7.2
κCN	YIPIQYVLSR	Extraction	1.14481E+12	5.72405E+11	6.4	2.8
		Digestion	2.99726E+12	4.99543E+11		38.5
		Ratio measurement	3.03071E+12	1.68373E+11		58.7
βLG	GLDIQK	Extraction	9,374,383,991	4,687,191,996	23.1	0.0
		Digestion	29,866,046,898	4,977,674,483		79.0
		Ratio measurement	5,269,055,283	585,450,587.1		21.0
	IPAVFK	Extraction	76,749,448,703	38,374,724,352	8.7	4.5
		Digestion	1.81446E+11	30,241,083,290		27.4
		Ratio measurement	2.46673E+11	13,704,048,151		68.1
	ALPMHIR	Extraction	4,533,019,326	2,266,509,663	9.6	0.0
		Digestion	34,287,299,411	5,714,549,902		2.0
		Ratio measurement	97,025,982,779	5,390,332,377		98.0
Ova	LYAEER*	Extraction	0.039886019	0.019943009	7.3	7.0
		Digestion	0.091391929	0.015231988		55.0
		Ratio measurement	0.051301231	0.002850068		38.0
	GLWEK	Extraction	3.13527E+11	1.56763E+11	11.8	0.1
		Digestion	9.37364E+11	1.56227E+11		70.8
		Ratio measurement	3.38734E+11	18,818,572,806		29.1
	HIATNAVLFFGR	Extraction	4.15205E+11	2.07602E+11	8.9	36.7
		Digestion	2.9878E+11	49,796,694,363		20.4
		Ratio measurement	3.68934E+11	20,496,340,998		42.9
Lys	GTDVQAWIR	Extraction	53,321,662,879	26,660,831,440	7.4	33.6
		Digestion	49,068,398,641	8,178,066,440		33.8
		Ratio measurement	35,821,898,572	1,990,105,476		32.6
	FESNFNTQATNR*	Extraction	0.006200414	0.003100207	6.5	0.0
		Digestion	0.022979051	0.003829842		72.5
		Ratio measurement	0.007724225	0.000429124		27.5
	HGLDNYR	Extraction	39,193,790.63	19,596,895.32	28.4	0.0
		Digestion	856,334,672.1	142,722,445.3		86.4
		Ratio measurement	127,696,469.5	7,094,248.306		13.6
Myo		Extraction	4.88984E+12	2.44492E+12	5.3	24.8
		Digestion	4.70281E+12	7.83802E+11		15.1
		Ratio measurement	8.04677E+12	4.47043E+11		60.1
Glu-Fib		Extraction	1.2567E+12	6.28352E+11	14	9.4
		Digestion	1.43487E+12	2.39144E+11		0.0
		Ratio measurement	3.42278E+12	2.0134E+11		90.6

*Peptides for which an isotope-labelled peptide standard was available

The results obtained from these studies suggest that, for the relatively high-level allergen contamination cookie studied, measurement precisions as low as 5% relative standard deviation (RSD) could be achieved. Where RSDs increase, normally due to the lack of an isotopic internal standard or a low measured signal intensity, digestion was the greatest source of variation in the majority of cases. The study suggests that while MS is capable of the multiplexed detection of many peptides, that a universal buffer which extracts all proteins equally is unlikely. Therefore, careful selection of quantitative marker proteins and robust peptides will be required.

## Conclusions

The ability of modern LCMS to sensitively and selectively detect the presence of allergen peptides has resulted in various multiplexed methods for the simultaneous detection of food allergens. However, when quantifying allergens, all parts of the analytical procedure need to be considered, not just the final MS method. While MS could quantify many peptides in one run, the ability to completely extract and digest the target marker proteins is essential if accurate results are to be obtained. The number of allergens that could be accommodated reasonably in a multiplexed assay depends on the following: the physicochemical properties and structure of the proteins; the complexity of the food matrix and its intermolecular interactions; and the food processing conditions (dough formation, baking, extrusion, and cooking/boiling).

The optimisation of protein extraction and digestion is fundamental to provide reliable quantification if proteotypic peptides are to be used for determining the amount of the allergenic food protein present. Current methods commonly use isotopically labelled peptides as internal standard. This approach is usually reducing the measurement bias and improving the precision of measurements at the peptide level. However, differences in extraction are often overlooked. The number of multiplexed MS methods currently being reported, where a single common buffer is used for the extraction of multiple proteins from many different allergenic food sources, suggests that MS has a lot to offer in the area of food allergen quantification. However, failure to fully address all aspects of the analytical procedure may result in unreported bias by the currently used approaches. The evidence presented in this study shows that the quantitative removal of proteins from heat-treated matrices is still a major challenge for multiplexed methods. Whilst multiplexed detection is possible, the accurate quantification of the total food allergen protein mass fraction will not be feasible without a universal buffer that extracts and dissolves the wide variety of proteins derived from the different allergenic food sources.

In this study, the developed method, specifically designed for the absolute quantification of egg and milk allergens in biscuits, required a compromised extraction and digestion protocol. No universal conditions were found that maximised the response of all the target peptides from milk and egg. Therefore, not all constituent proteins can be considered good quantitative markers and should simply be used as qualitative markers. Knowing the digestion kinetics delivered evidence that peptides were representative of the protein content, thereby providing confidence in the determined concentration for each protein. With the inclusion of labelled peptide internal standards, the precision of the MS peak area ratio measurements had very little impact on the overall variability of the analytical result. Therefore, tighter control of the digestion and extraction procedures is essential for achieving improved measurement uncertainties in the future.

## Electronic supplementary material

ESM 1 (PDF 2496 kb)
