# Supplementary material for: An assessment of the impact of extraction and digestion protocols on multiplexed targeted protein quantification by mass spectrometry for egg and milk allergens
Source: Anal Bioanal Chem. 2019 May 28;411(16):3463–75. doi: 10.1007/s00216-019-01816-z (PMC6571087; doi:10.1007/s00216-019-01816-z)
Supplement: Supplementary file 1 — (PDF 2496 kb) [file 216_2019_1816_MOESM1_ESM.pdf]

Analytical and Bioanalytical Chemistry

Electronic Supplementary Material

An assessment of the impact of extraction and digestion protocols on multiplexed-targeted protein quantification by mass spectrometry for egg and milk allergens

Chiara Nitride, Jørgen Nørgaard, Jone Omar, Hendrik Emons, María-José Martínez Esteso, Gavin O'Connor

S1 Recipe for the cookies analysed for milk and to make 1 kg of cookie dough

Ingredients

Peanut flour 10.8 g
Hazelnut flour 19.5 g
Skimmed milk powder (SMP) 14.4 g
Instant egg white powder (EWP) 7 g
Biscuit flour 481 g
Bicuit fat 168.6 g
Icing sugar 154.4 g
Salt 5.5 g
Sodium bicarbonate 1.8 g
Ammonium bicarbonate 1.1 g
Water 135.9 g

Preparation

The biscuit fat and icing sugar were first mixed until it formed a smooth paste. The sodium bicarbonate, ammonium bicarbonate and salt were added to the water. The solution was then added to the sugar/fat paste. The peanut flour, hazelnut four, SMP and EWP were thoroughly mixed with 48 g of the biscuit flour. Once thoroughly mixed the remaining flour was added and mixed thoroughly. The flour was slowly added to water paste and mixed using paddle mixer until a dough was formed.

The resulting dough was rolled to an even thickness of 1.5 mm and was then cut into 2.54 cm squares. The biscuits were placed on greaseproof paper and backed in an oven for 9 min at 150 °C and 10% humidity.

Fig. S1 Flowchart representing the buffering systems, the chaotropics and reducing agents used in the extraction screening

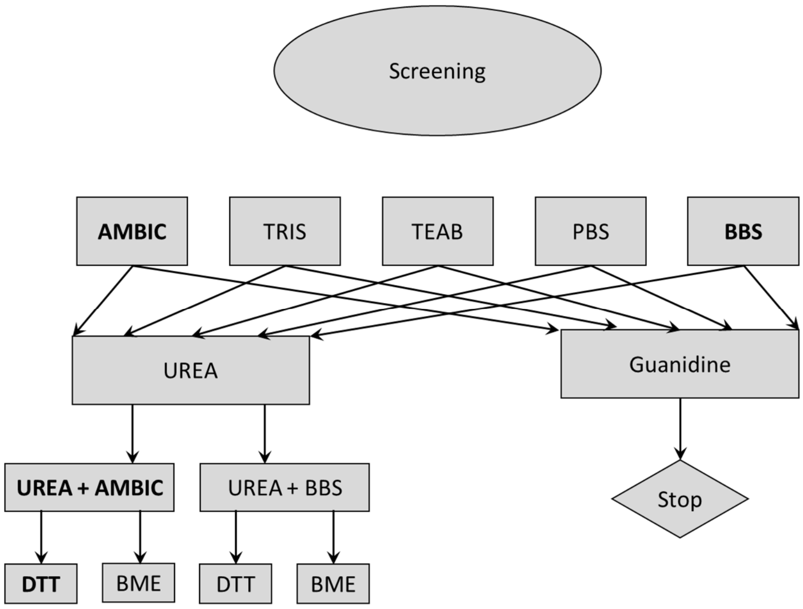

Fig. S2 Combined SRM chromatograms for the selected milk and egg peptides from a trqptic digestion of a cookie extract

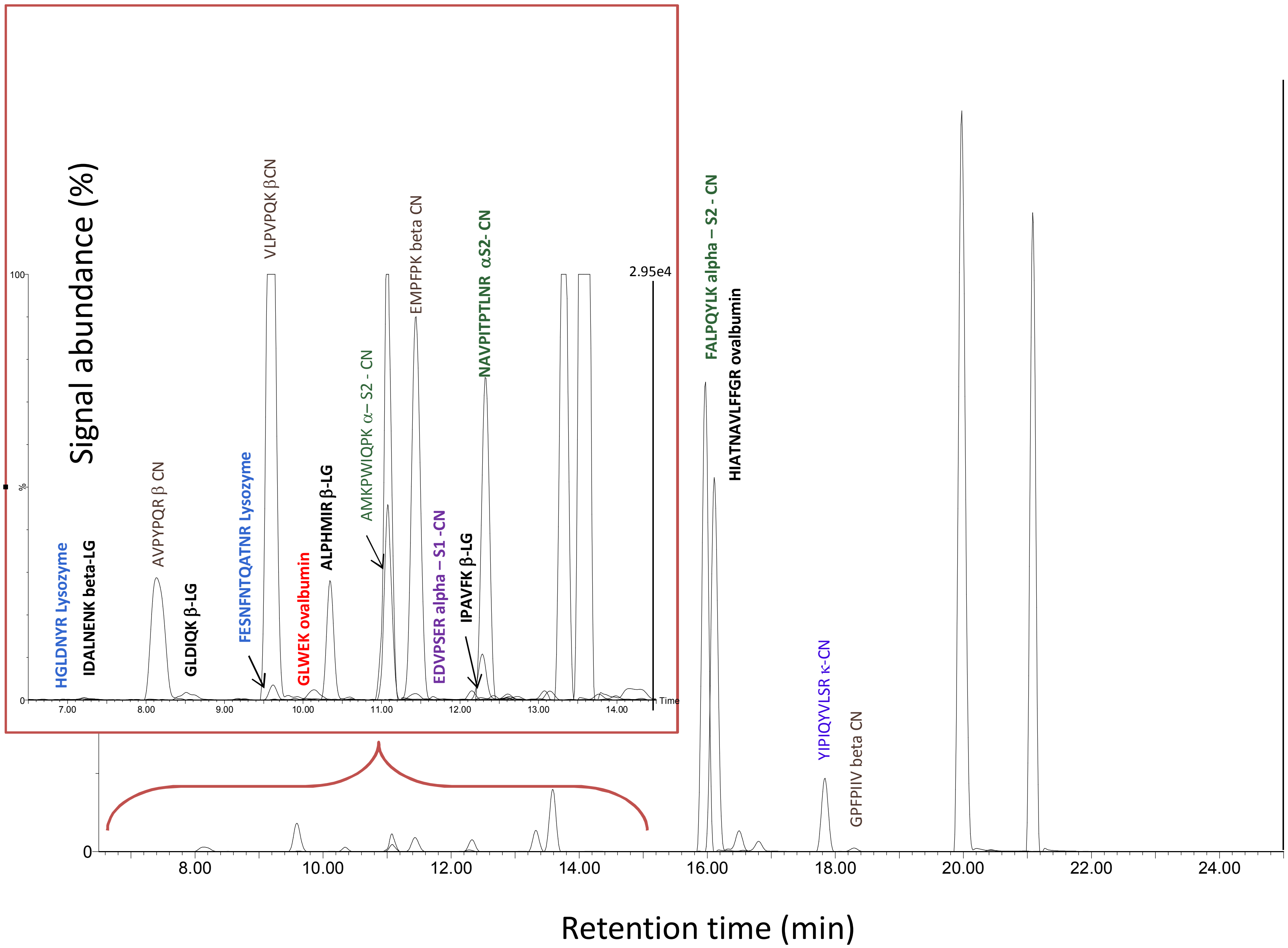

Fig. S3 Graphic comparison of the estimated total protein concentration (expressed as µg of total protein per mL of extract) determined using the RCDC™ protein assay. (a) Extraction with buffer only, (b) extraction with chaotropic agent, (c) extraction with chaotropic and reducing agent. Error bars represent the standard deviation of 3 replicate measurements

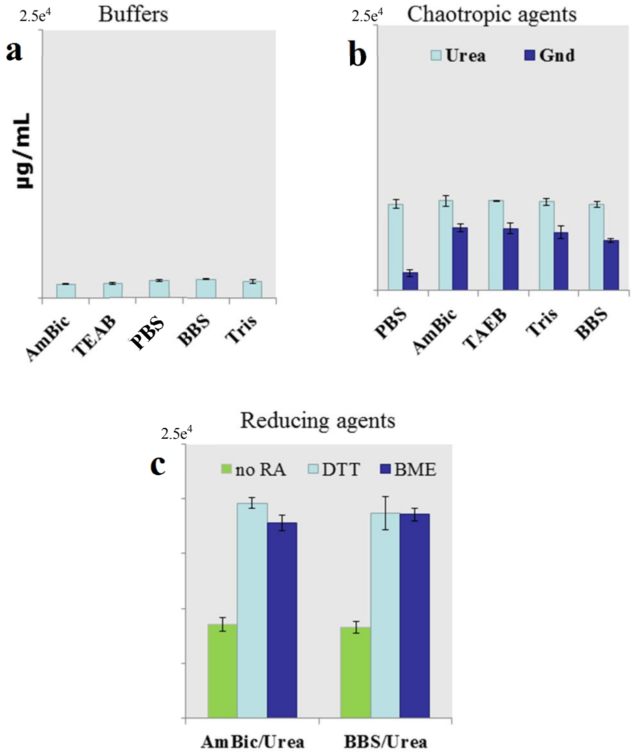

Fig. S4 Graph comparison of the SRM peak area ratios of the milk derived peptides released after digesting proteins extracted from the biscuits using two chaotropic agents ☐ Guanidine and ■ Urea. Error bars represent the standard deviation of 3 replicate measurements

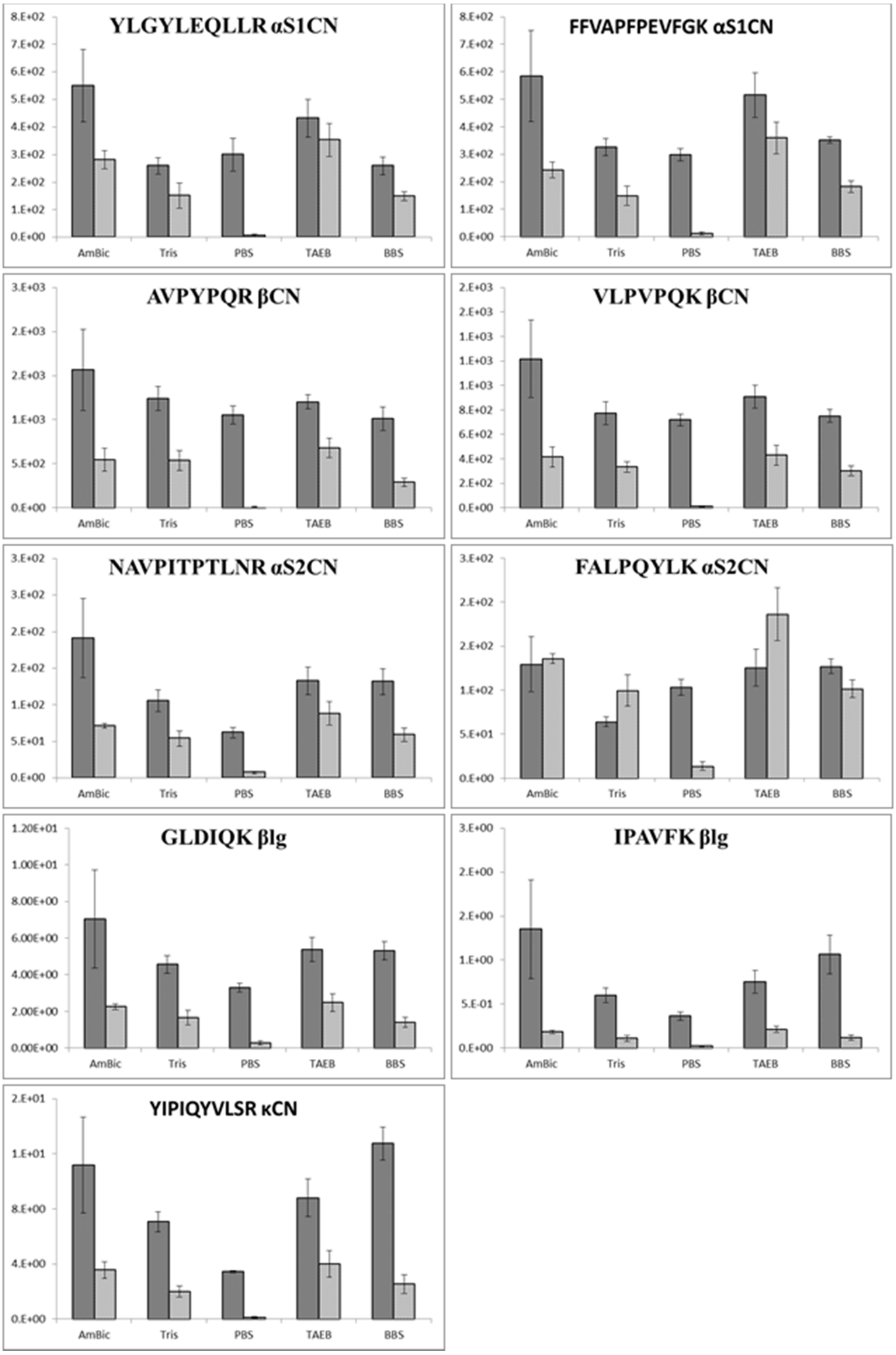

Fig. S5 Graph comparison of the SRM peak areas of the egg white derived peptides released after digesting proteins extracted from the biscuits using two chaotropic agents ☐ Guanidine ■ Urea. Error bars represent the standard deviation of 3 replicate measurements

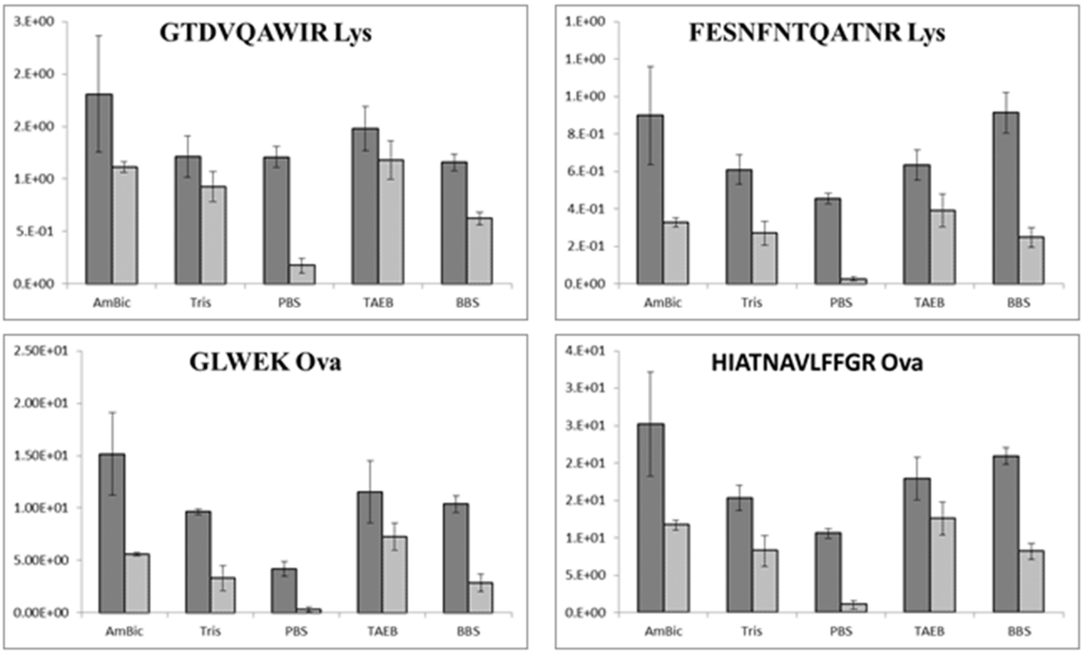

Fig. S6 Graph comparison of the SRM peak areas of the milk derived peptides released after digesting proteins extracted from the biscuits ☐ w/o reducing agent and with ■ 5% DTT or with ■ 5% BME. Error bars represent the standard deviation of 3 replicate measurements

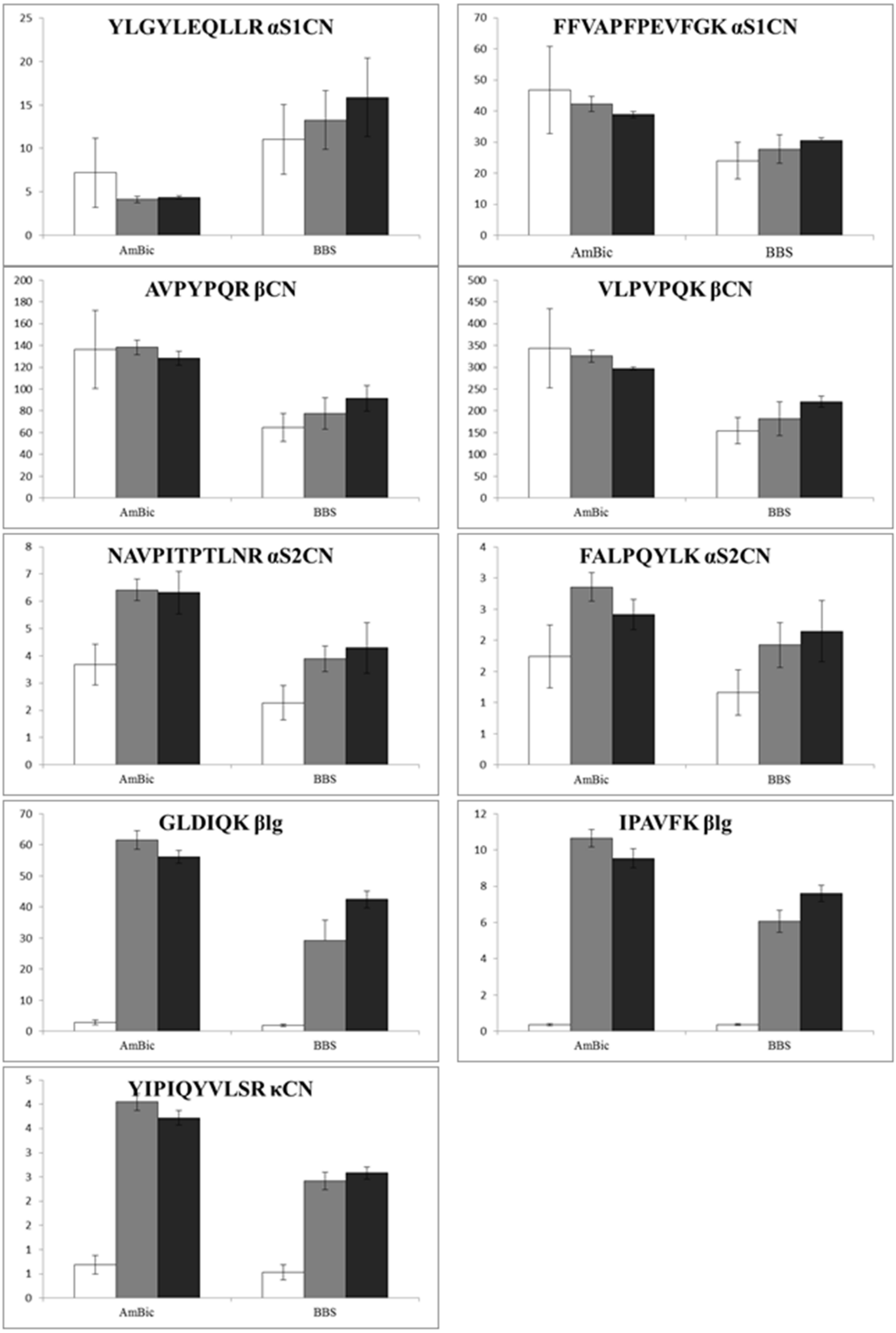

Fig. S7 Graph comparison of the SRM peak areas of the egg white derived peptides released after digesting proteins extracted from the biscuits ☐ w/o reducing agent and with ■ 50 mmol L^{-1} DTT or with ■ 5% BME. Error bars represent the standard deviation of 3 replicate measurements

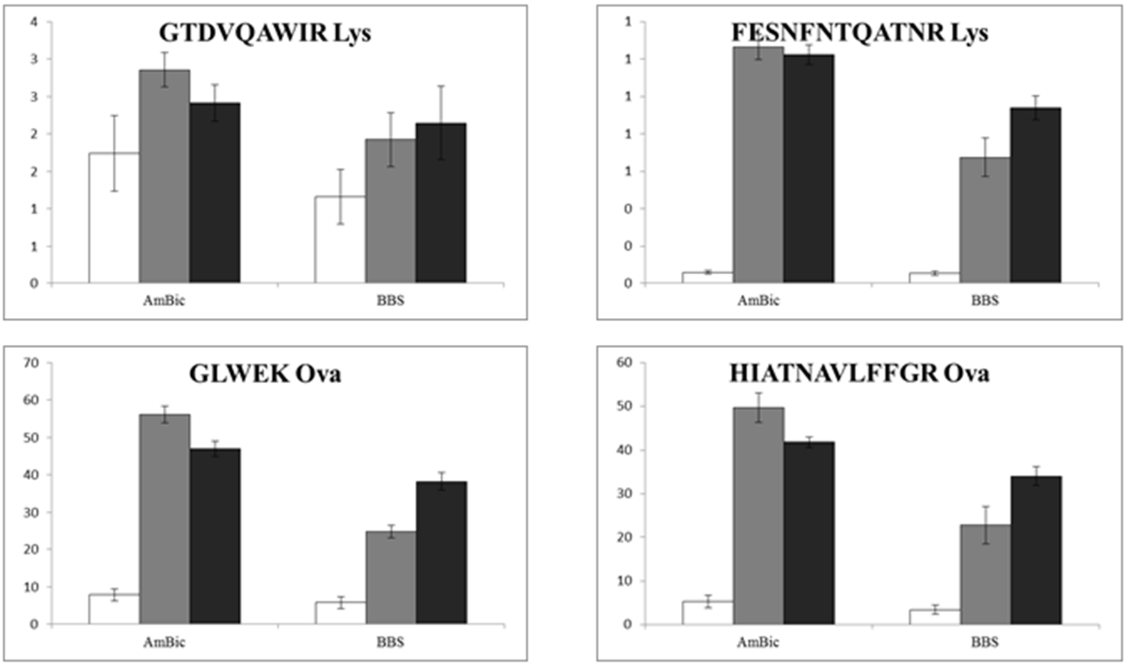

Fig. S8 Flowchart used for the optimisation of extraction and digestion protocols

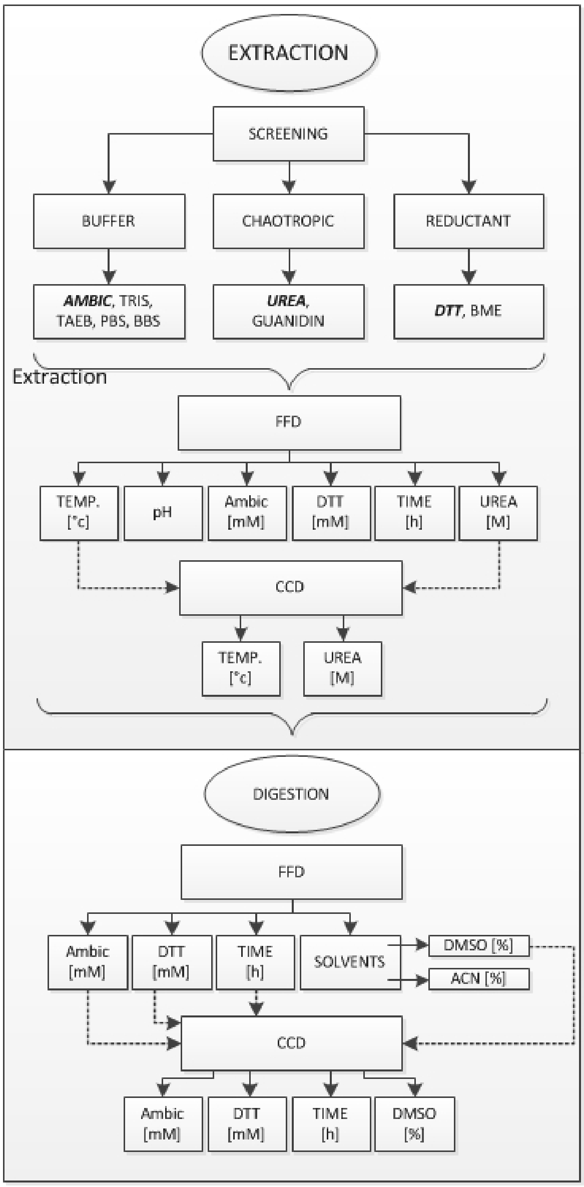

Fig. S9 Surface plots showing the effects of Urea concentration and temperature on the extractability of the proteins. The protein concentration (expressed as µg of total protein extracted from 0.5 g of biscuits) was estimated using the RCDC™ protein assay

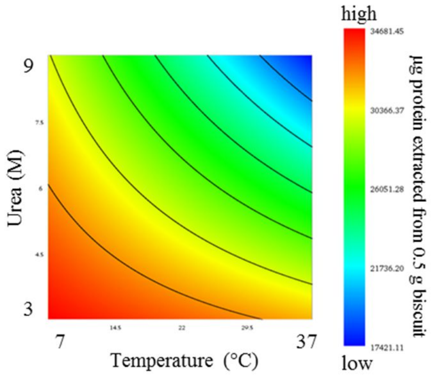

Fig. S10 Surface plots showing the effects of Urea concentration (B) and temperature (A) (p-level < 0.05) on the peak area of the peptides. Panel a) β LG-IPAVFK; b) β CN-VLPVPQK; c) Ova – GLWEK; d) Ova – LYAEER; e) Ova - VASMASEK; f) Lys- HGLD-NYR; g); Lys- GTDVQAWIR

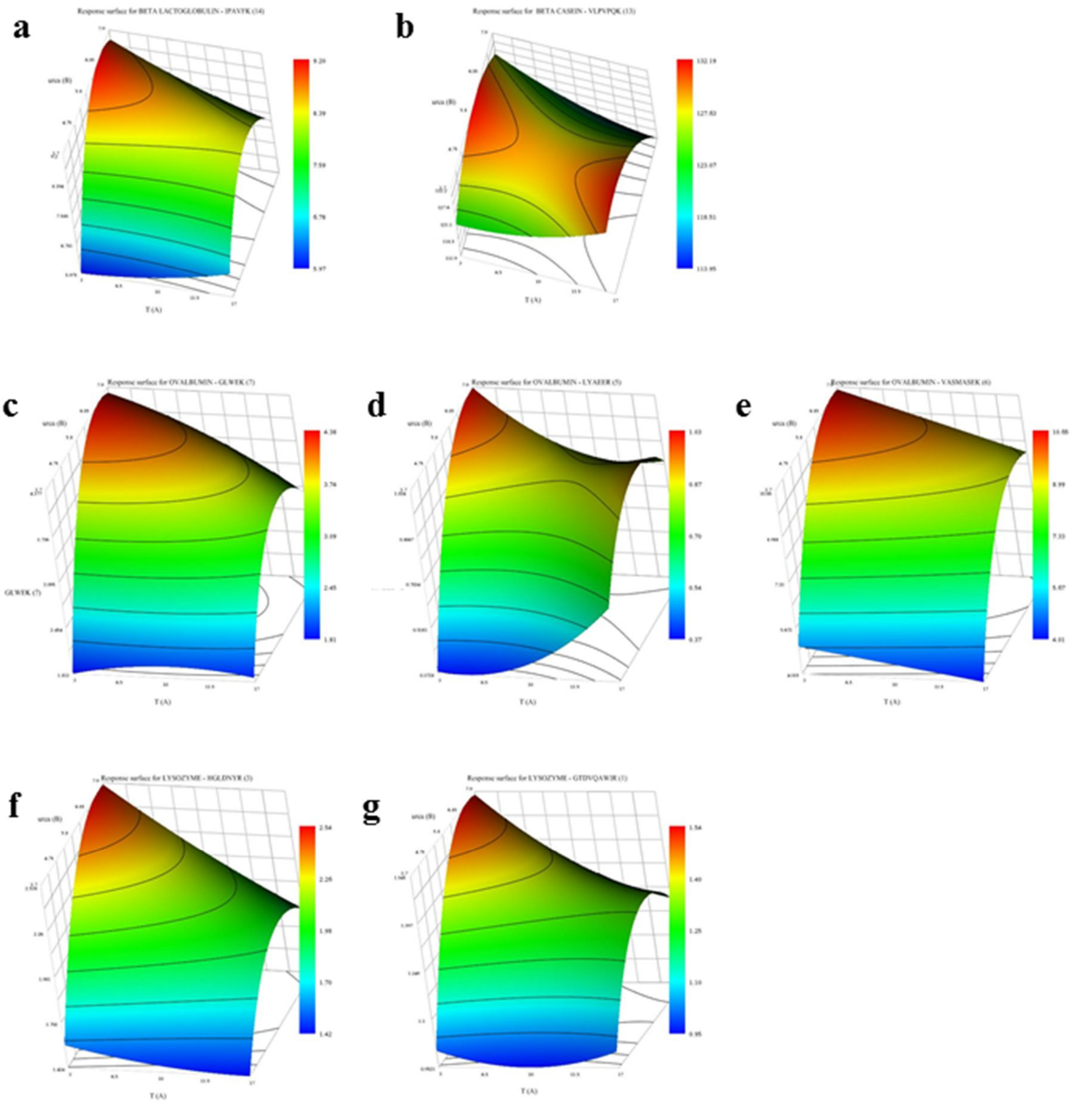

Fig. S11 Surface plots showing the effects of the statistically significant variables (p-level < 0.05) on the peak area of the peptides. Panel a) α S2 CN- ALNEINQFYQK; c) α S1 CN-YLGYLEQLLR; d) β CN-VLPVPQK; e) Ova- GLWEK; f) Ova- HIATNAVLFFGR; g) Lys-FESNFNTQATNR

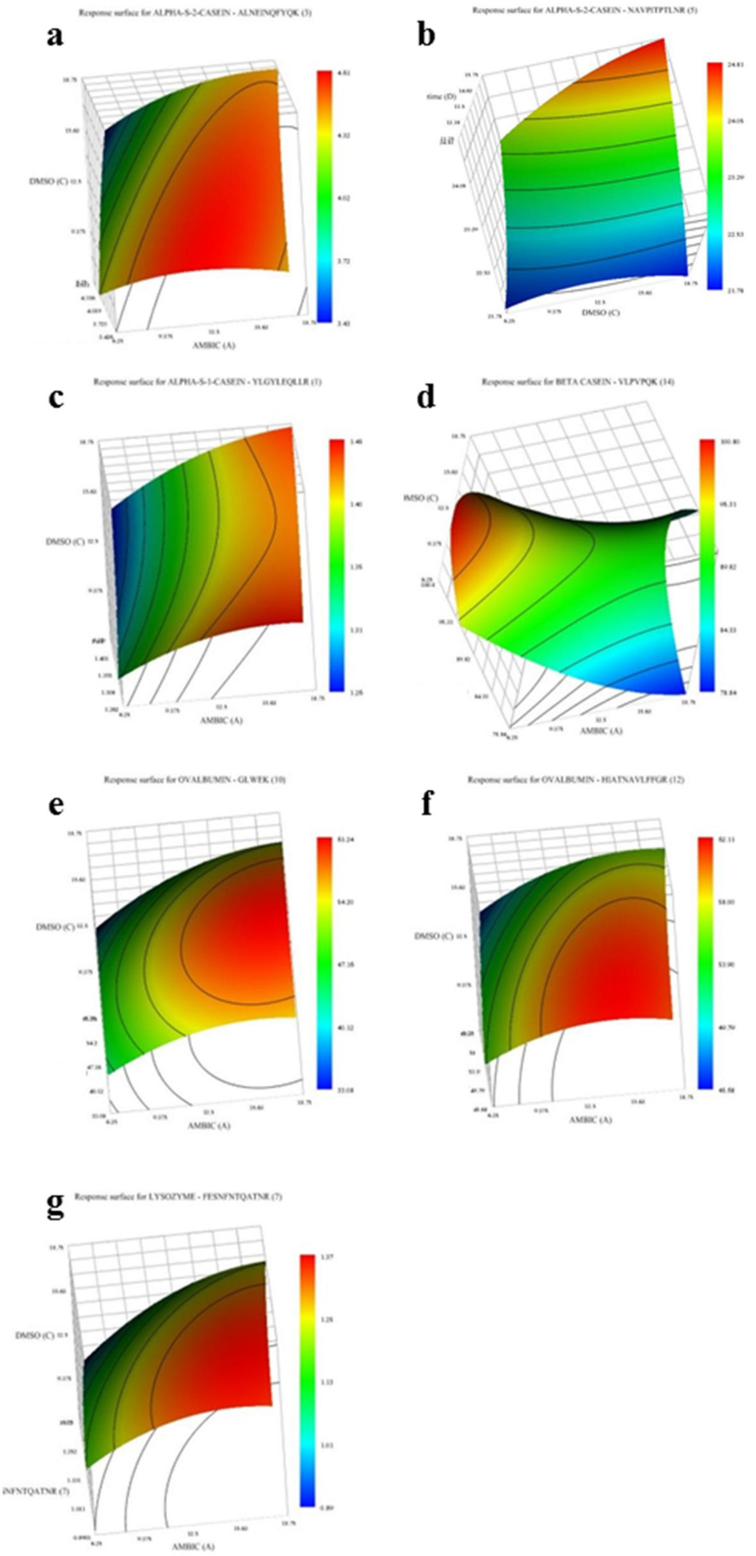

Fig. S12 Profile of marker peptides released under optimised digestion conditions over 35 hours of digestion. The addition of trypsin was static and multiple. Static addition of trypsin was perform only at time 0 hours at two E:S ratios - 1:50 and 1:100. Multiple additions of trypsin were performed at equidistant intervals of time to a final 1:25 (E:S) ratio - 1:50 (E:S) ratio was added at time 0 and time 22 hours; 1:100 (E:S) ratio was added at time 0, 6, 12 and 22 hours. Error bars represent the standard deviation of 3 replicate measurements. Panel a) α S2 CN- NAVPITPTLNR; b) α S2 CN- ALNEINQFYQK; c) α S2 CN- VIPYVR; d) β LG- IPAVFK; e) β LG- ALPMHIR; f) β LG- GLDIQK; g) α S1 CN- FFVAPFPEVFGK; h) κ CN - YIPIQYVLSR ; i) β CN- VLPVPQK ; j) β CN- AVPYPQR; k) Ova- GLWEK; l) Ova- HIATNAVLFFGR; m) Lys- HGLDNYR; n) Lys- GTDVQAWIR

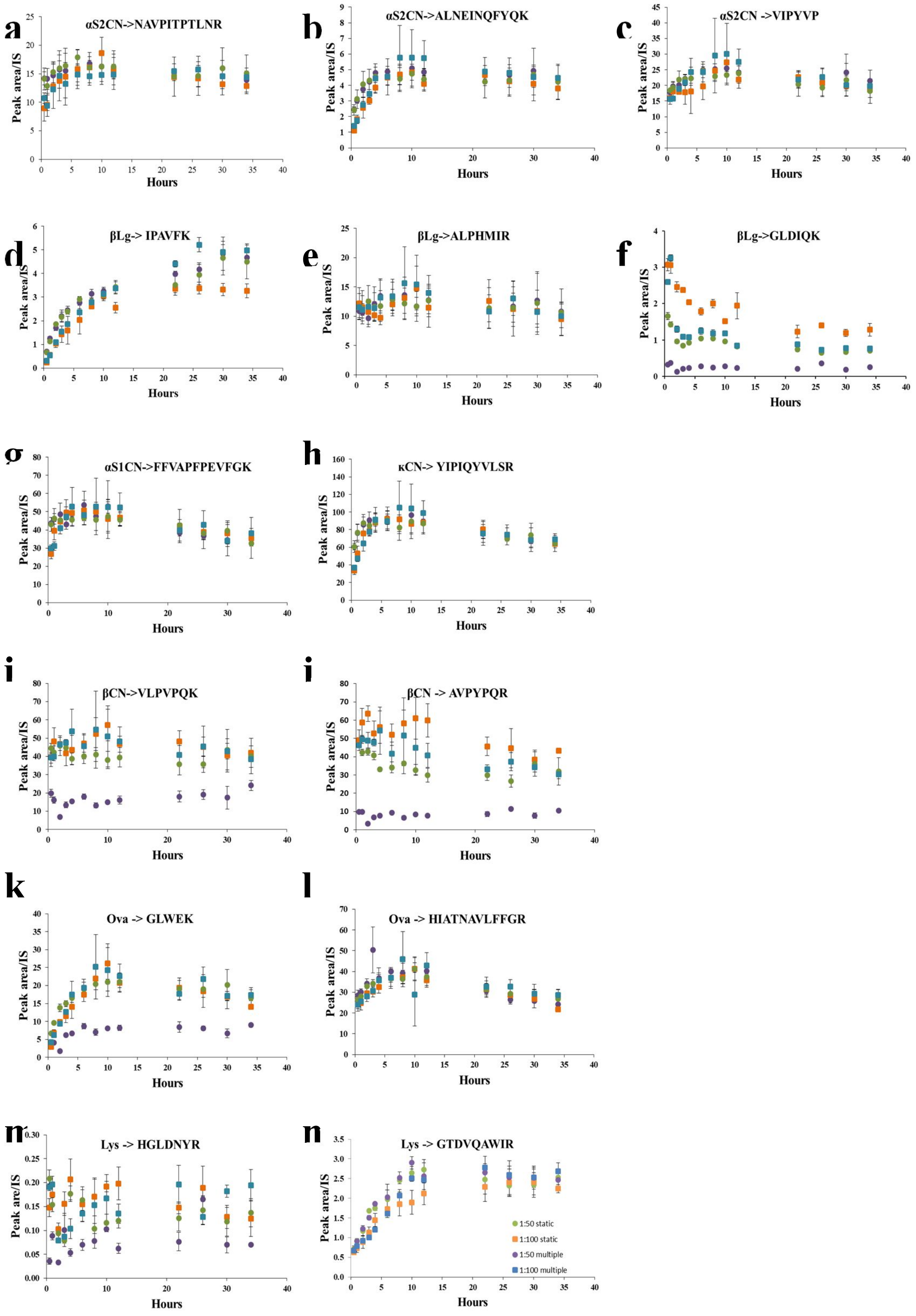

Table S1 List of MS-Homology blast hits that produce significant alignments with our query sequences

Protein	Peptide	Uniqueness	BLAST
αS1	EDVPSER	Milk	P02662 O62823 P18626 P04653
	YLGYLEQLLR	Milk	P02662 B6ZBP2 D3TU01 Q69EZ6
	FFVAPFPEVFGK	Milk	P02662 Q4F6X6 Q865A0 D3TJU0
αS2	NAVPITPTLNR	Cow Milk	P02663
	AMKPWIQPK	Cow Milk	P02663
	FALPQYLK	Cow Milk	P02663
	VIPYVR	No	P02663 Q9FS88 O74773 A3QK15
	ITVDDK	No	P02663 P33049 P04654 P23458 P34544 B4SJW7
	ALNEINQFYQK	Milk	P02663 P33049 P04654
βCn	AVPYPQR	Milk	P02666 Q9TSI0
	EAMAPK	No	P02666 Q9TSI0 Q2HGJ1
	EMPFPK	Milk	P02666 Q9TSI0 P33048 P11839
	VLPVPQK	Milk	P02666 Q9TSI0 P33048 P11839
	GPFPIIV	No	P02666 Q9TSI0 T0IJL6 W4EZJ2 D2QIT4
βLg	IDALNENK	Milk	P02754 P02755 P02756 P67975 P67976
	GLDIQK	Cow Milk	P02754
	ALPMHIR	Milk	P02754 P02755 P02756 P67975 P67976
	IPAVFK	Milk	P02754 P02755 P02756 P67975 P67976
	TPEVDDEALEK	Milk	P02754 P02755
κCn	YIPIQYVLSR	Milk	P02668 P11840 P42156 P02670 P50420 P50421
Ova	LYAEER	Hen Egg	P01012
	GLWEK	No	P01012 G1MYK6 P19104 E2RVI8
	VASMASEK	Egg	P01012 Q6V115 P19104 O73860
	HIATNAVLFFGR	Hen Egg	P01012
Lys	HGLDNYR	Egg	P00698 P00699 P00700 P00704
	FESNFNTQATNR	Egg	P00698 P00701
	GTDVQAWIR	Egg	P00698 P00700 Q7LZI3

Table S2 Overview of 19 peptides from the 5 cow milk proteins and two hen egg white proteins, of the product ions, cone voltage (V) and collision energies (eV) used for the SRM

Protein	Peptide	MW (Da)	Parent (Da)	Daughter (Da)		Cone (V)	Coll (eV)
α S1 CN	YLGYLEQLLR	1267.7	634.4	334.2	b3	25	20
				771.5	y6		18
				991.6	y8		20
	FFVAPFPEVFGK	1384.7	692.9	465.2	b4	25	12
				920.5	y8		18
				991.5	y9		18
α S2 CN	VIPYVR	746.5	373.7	437.3	y3	15	15
				534.3	y4		8
	FALPQYLK	979.6	490.3	648.4	y5	15	12
				761.5	y6		12
	NAVPITPTLNR	1195.7	598.3	285.2	b3	15	15
				911.5	y8		15
	ALNEINQFYQK	1367.7	684.4	428.2	b4	15	20
				827.4	y6		20
β CN	VLPVPQK	780.5	390.8	275.2	y2	20	20
				372.2	y3		15
				568.3	y5		8
	AVPYPQR	830.5	415.7	400.2	y3	20	15
				563.3	y4		15
				660.3	y5		10
κ CN	YIPIQYVLSR	1251.7	626.4	637.4	y5	25	20
				765.4	y6		20
				975.6	y7		20
β LG	GLDIQK	673.4	337.2	388.3	y3	25	10
				503.3	y4		8
				527.3	b6		10
	IPAVFK	674.4	337.7	294.2	y2	25	15
				393.2	y3		12
				464.3	y4		10
				561.3	y5		10
	ALPMHIR	837.5	419.2	327.2	y5+2	26	8
				425.3	y3		15
				556.3	y4		15
				653.4	y5		12
Ova	LYAEER	780.4	390.7	304.2	y2	12	18
				504.2	y4		10
				667.3	y5		12
	GLWEK	632.3	316.7	276.2	y2	10	12
				462.2	y3		8
	HIATNAVLFFGR	1345.7	449.3	526.3	y4	22	8
				608.3	b6		10
				639.4	y5		10
	VASMASEK	823.0	411.7	434.2	y4	14	12
				565.3	y5		10
Lys	GTDVQAWIR	1045.5	523.3	474.3	y3	20	15
				545.3	y4		15
				673.4	y5		15
	FESNFNTQATNR	1428.7	714.8	461.2	y4	25	20
				589.3	y5		20
				690.4	y6		22
	HGLDNYR	874.4	437.7	452.2	y3	25	18
				680.3	y5		15
				737.4	y6		12
**Protein**	**Labelled Peptide**	**MW (Da)**	**Parent (Da)**	**Daughter (Da)**		**Cone (V)**	**Coll (eV)**
α S1 CN	YLGYLEQLL**R***	1276.7	639.4	1001.6		25	20
α S2 CN	FALPQYL**K***	984.6	494.4	656.2		15	12
Ova	LYAEE**R***	789.4	395.7	514		12	18
Lys	FESNFNTQATN**R***	1437.7	719.8	471.1		25	20

Table S3 Individual p-values for the Full Factorial Design of the extraction

	**αS2CN-VIPYVP**	**αS2CN-ALNEINQFYQK**	**αS2CN-NAVPITPTLNR**	**αS2CN-FALPQYLK**
AmBic (mM)				
pH				
T (°C)	0.0006	0.0179	0.0324	0.0037
Urea (M)		0	0	0.0900
Time (h)				
DTT (mM)				
	**βLG-IPAVFK**	**βLG-ALPHMIR**	**βLG-GLDIQK**	
AmBic (mM)				
pH				
T (°C)	0.0189	0.0081	0.0044	
Urea (M)		0.0054	0.0028	
Time (h)				
DTT (mM)				
	**αS1CN-FFVAPFPEVFGK**	**αS1CN-YLGYLEQLLR**		
AmBic (mM)				
pH				
T (°C)	0.0629			
Urea (M)	0	0.0692		
Time (h)				
DTT (mM)				
	**βCN-AVPYPQR**	**βCN-VLPVPQK**		
AmBic (mM)				
pH				
T (°C)	0.0445	0.0255		
Urea (M)	0	0		
Time (h)				
DTT (mM)				
	**κ-CN-YIPIQYVLSR**			
AmBic (mM)				
pH				
T (°C)				
Urea (M)	0			
Time (h)				
DTT (mM)				
	**Ova-GLWEK**	**Ova-HIATNAVLFFGR**	**Ova-LYAEER**	
AmBic (mM)				
pH				
T (°C)				
Urea (M)	0	0.0001	0.0051	
Time (h)				
DTT (mM)				
	**Lys-HGLDNYR**	**Lys-FESNFNTQATNR**	**Lys-GTDVQAWIR**	
pH				
AmBic (mM)				
T (°C)		0.0217	0.0095	
Urea (M)	0.0008	0	0.0006	
Time (h)				
DTT (mM)				

Table S4 Individual p-values for the Central Composite Design of the extraction. *interaction - Indication of the quadratic effect of the Urea concentration

	**αS2CN-VIPYVP**	**αS2CN-ALNEINQFYQK**	**αS2CN-NAVPITPTLNR**	**αS2CN-FALPQYLK**
T (°C)				
Urea (M)				
	**βLG-IPAVFK**	**βLG-ALPHMIR**	**βLG-GLDIQK**	
T (°C)				
Urea (M)	0.0055	0.0009		
	**αS1CN-FFVAPFPEVFGK**	**αS1CN-YLGYLEQLLR**		
T (°C)				
Urea (M)	0.0071			
	**βCN-AVPYPQR**	**βCN-VLPVPQK**		
T (°C)				
Urea (M)				
		B*B interaction		
		0.0032		
	**κ-CN-YIPIQYVLSR**			
T (°C)				
Urea (M)				
	**Ova-GLWEK**	**Ova-HIATNAVLFFGR**	**Ova-LYAEER**	
T (°C)	0.0410	0.0122		
Urea (M)	0.0001	0	0.0472	
	**Lys-HGLDNYR**	**Lys-FESNFNTQATNR**	**Lys-GTDVQAWIR**	
T (°C)	0.0044	0.0006	0.0322	
Urea (M)	0.0003	0	0.0005	

Table S5 Individual p-values for the Full Factorial Design of the digestion

	**αS2CN-VIPYVP**	**αS2CN-ALNEINQFYQK**	**αS2CN-NAVPITPTLNR**	**αS2CN-FALPQYLK**
Time (h)		0.094871	0.041105	
AmBic (mM)	0.028126		0.000520	
DTT (mM)		0.028083	0.002752	
AcN (%)		0.012932		0.009966
DMSO (%)	0.075594		0.009483	
	**βLG-IPAVFK**	**βLG-ALPHMIR**	**βLG-GLDIQK**	
Time (h)	0.014354			
AmBic (mM)	0.068737			
DTT (mM)	0.010727	0.034035		
AcN (%)	0.021182			
DMSO (%)				
	**αS1CN-FFVAPFPEVFGK**	**αS1CN-YLGYLEQLLR**		
Time (h)				
AmBic (mM)				
DTT (mM)	0.026569			
AcN (%)	0.014339	0.007933		
DMSO (%)				
	**βCN-AVPYPQR**	**βCN-VLPVPQK**		
Time (h)				
AmBic (mM)	0.033713			
DTT (mM)				
AcN (%)				
DMSO (%)		0.312077		
	**κCN-YIPIQYVLSR**			
Time (h)				
AmBic (mM)	0.000631			
DTT (mM)				
AcN (%)				
DMSO (%)				
	**Ova-GLWEK**	**Ova-HIATNAVLFFGR**	**Ova-LYAEER**	
Time (h)	0.014089	0.000003		
AmBic (mM)	0.002194	0.000555		
DTT (mM)	0.010079	0.019807		
AcN (%)		0.000100		
DMSO (%)	0.026843			
	**Lys-HGLDNYR**	**Lys-FESNFNTQATNR**	**Lys-GTDVQAWIR**	
Time (h)		0.001023	0.015481	
AmBic (mM)		0.090525	0.067819	
DTT (mM)		0.000329	0.020531	
AcN (%)				
DMSO (%)		0.001909	0.071218	

Table S6 Individual p-values for the central composite design of the digestion. *interaction - Indication of the quadratic effect of the factor A= AmBic (mM); C=DMSO (%)

	**αS2CN-VIPYVP**	**αS2CN-ALNEINQFYQK**	**αS2CN-NAVPITPTLNR**	**αS2CN-FALPQYLK**
AmBic (mM)		0		
DTT (mM)				
DMSO (%)		0.0026		
Time (h)		0.0020	0.0068	
		A*C		
	**βLG-IPAVFK**	**βLG-ALPHMIR**	**βLG-GLDIQK**	
AmBic (mM)				
DTT (mM)				
DMSO (%)			0.0077	
Time (h)				
	**αS1CN-FFVAPFPEVFGK**	**αS1CN-YLGYLEQLLR**		
AmBic (mM)		0.0015		
DTT (mM)				
DMSO (%)				
Time (h)				
	**βCN-AVPYPQR**	**βCN-VLPVPQK**		
AmBic (mM)				
DTT (mM)				
DMSO (%)	0.001500			
Time (h)				
	C*C	C*C		
	**κCN-YIPIQYVLSR**			
AmBic (mM)				
DTT (mM)				
DMSO (%)				
Time (h)				
	**Ova-GLWEK**	**Ova-HIATNAVLFFGR**	**Ova-LYAEER**	
AmBic (mM)	0.0071	0.0024	0.0000	
DTT (mM)				
DMSO (%)			0.0000	
Time (h)			0.0000	
	C*C		C*C	
	**Lys-HGLDNYR**	**Lys-FESNFNTQATNR**	**Lys-GTDVQAWIR**	
AmBic (mM)		0.0048	0.0002	
DTT (mM)				
DMSO (%)				
Time (h)			0.0038	
